# Longitudinal evaluation of manufacturer-specific differences for high-sensitive CRP EQA results

**DOI:** 10.3389/fmolb.2024.1401405

**Published:** 2024-08-08

**Authors:** Nathalie Weiss, Laura Vierbaum, Marcel Kremser, Anne Kaufmann-Stoeck, Silke Kappler, Silvia Ballert, Kathrin Kabrodt, Klaus-Peter Hunfeld, Ingo Schellenberg

**Affiliations:** ^1^ INSTAND e.V., Society for Promoting Quality Assurance in Medical Laboratories e.V., Duesseldorf, Germany; ^2^ Institute of Bioanalytical Sciences (IBAS), Center of Life Sciences, Anhalt University of Applied Sciences, Bernburg, Germany; ^3^ Medical Faculty, Northwest Medical Centre, Academic Teaching Hospital, Institute for Laboratory Medicine, Microbiology and Infection Control, Goethe University Frankfurt, Frankfurt, Germany

**Keywords:** hsCRP, external quality assessment scheme (EQA), proficiency testing (PT), harmonization, cardiovascular diseases

## Abstract

**Background:**

C-reactive protein (CRP) is an established serum biomarker for different pathologies such as tissue injury and inflammatory events. One rising area of interest is the incorporation of low concentrations of CRP, so called high-sensitive (hs-) CRP, in the risk assessment and treatment monitoring of cardiovascular diseases (CVDs). Many research projects and the resulting meta-analyses have reported controversial results for the use of hs-CRP, especially in the risk assessment of CVDs. However, since these analyses used different assays to detect hs-CRP, it is important to assess the current level of assay harmonization.

**Methods:**

This paper analyzes data from 17 external quality assessment (EQA) surveys for hs-CRP conducted worldwide between 2018 and 2023. Each EQA survey consisted of two blinded samples. In 2020 the sample material changed from pooled serum to single-donor samples. The aim was to assess the current status of assay harmonization by a manufacturer-based approach, taking into consideration the clinical decision limits for hs-CRP risk-stratification of CVDs as well as the scatter of results.

**Results:**

Our analyses show that harmonization has increased in recent years from median differences of up to 50% to below 20%, with one exception that showed an increasing bias throughout the observed period. After changing sample materials from pools to single-donor samples, the coefficient of variation decreased to below 10% with one exception. Nevertheless, even these differences in the clinical setting could lead to disparate classification of patients depending on the assay used.

**Conclusion:**

While there was a positive trend towards harmonization, meta-analysis of different risk-score publications should stratify their analysis by assay to account for the manufacturer-specific differences observed in this paper. Furthermore, assays are currently traceable to different international standard preparations, which might have a negative impact on future harmonization.

## 1 Introduction

C-reactive protein (CRP) is an acute-phase protein that is predominantly produced in hepatocytes in response to tissue injury, inflammatory events, acute infection and advanced age (Póvoa et al., 1998; Póvoa, 2002; Pepys and Hirschfield, 2003; Lobo, 2012). After more and more evidence emerged, that cardiovascular diseases (CVDs) such as ischemic stroke and acute myocardial infarction are related to inflammation (Libby et al., 2002), moderately elevated levels of CRP, so called high-sensitive CRP (hs-CRP), gained interest as a potential new biomarker for these diseases. This need was further highlighted by the fact that CVDs account for 17.9 million deaths annually (WHO, 2023). In Germany, they show a rising prevalence (Heidemann et al., 2021) and cost the German healthcare system €56.4 billion, around 13.1% of all German healthcare costs (Statistisches Bundesamt, 2022). Internationally, CVDs are estimated to have cost €282 billion in 2021in the European Union (European Society of Cardiology, 2023), and $219 billion (∼€185 billion) in the United States (CDC, 2021). A systematic review of 49 cost-effectiveness studies concluded that early CVD detection and treatment was predominately cost-effective from a healthcare perspective, but it was also noted a lack of standardization in the included studies (Oude Wolcherink et al., 2023). Nevertheless, high hopes were placed on new markers, that allow an early detection of CVD-risk.

While hs-CRP is commonly used in clinical practice as an inflammatory marker in CVD risk assessments (Musunuru et al., 2008; Romero-Cabrera et al., 2022), its actual clinical significance remains controversial. Several meta-analyses show a positive effect of using hs-CRP to detect CVDs (Li et al., 2017; Romero-Cabrera et al., 2022). Further studies support a beneficial outcome for incidences of CVD events when hs-CRP is included in treatment decisions for CVDs (Ridker et al., 2008; Ridker et al., 2017). However, other authors found no or only marginal evidence for an improvement of hs-CRP-supported CVD risk stratification using scoring systems (Shah et al., 2009) and a low predictive utility of hs-CRP (Ahmad et al., 2024). A systematic review by the U.S. Preventive Services Task Force concluded that, based on the studies it reviewed, incorporating hs-CRP in risk stratification would lead to more misclassified individuals and thus overtreatment. It concluded that there is a lack of significantly conclusive clinical trials that evaluate the incremental effect of hs-CRP and other cardiovascular markers for the initiation of preventive therapy (Lin et al., 2018). Due to the inconclusive results, several international clinical guidelines are currently advising against incorporating hs-CRP in the corresponding risk-assessment algorithms (Piepoli et al., 2016; Deutsche Gesellschaft für Allgemeinmedizin und Familienmedizin e.V., 2017; Visseren et al., 2022; Australian Chronic Disease Prevention Alliance, 2023).

One interesting factor that arises is that meta-analyses, like the one performed by Li et al., aggregate data from various publications in which hs-CRP results were obtained using different assays. It is therefore important that the analytical performance of these various diagnostic tests be reliable and, ideally, harmonized regardless of the measurement procedure used. This would allow a more efficient comparison of the results of different CVD studies with respect to the diagnostic properties of hs-CRP. Even though a certified reference material for CRP exists (Charoud-Got et al., 2009), several studies have reported manufacturer-dependent differences for (hs-)CRP detection in serum (Roberts et al., 2000; Roberts et al., 2001; Thanabalasingham et al., 2011; Wojtalewicz et al., 2019; Stevenson et al., 2023).

This study examines the status of current assay-harmonization for hs-CRP based on the longitudinal manufacturer-dependent differences observed in EQA surveys conducted by INSTAND e.V. – Society for Promoting Quality Assurance in Medical Laboratories e.V. between 2018 and 2023.

## 2 Materials and methods

### 2.1 Sample materials—properties and preparation

From January 2018 until July 2020, commercially pooled serum samples of 1 mL were used. Starting in September 2020, the material was changed to 0.3 mL samples, mostly from individual blood donors. No stabilizing additives were added (Müller et al., 2009). This study was conducted in accordance with the Statement of the Central Ethics Commission of Germany on the use of human body materials for medical research purposes (no. 20/02/2003; https://www.zentrale-ethikkommission.de). The donor’s informed written consent is available for the single donor samples of the project. A positive vote from the ethics committee of Goethe University Frankfurt (Main) has been obtained for samples from voluntary blood donors.

All samples (pools and individual donors) tested negative for HIV, HBV, and HCV. Homogeneity of each sample batch was tested in line with DIN EN ISO/IEC 17043:2023 before the samples were used in the corresponding EQA (International Organization for Standardization [ISO], 2023).

### 2.2 EQA procedure

The INSTAND EQA scheme for the detection of hs-CRP in serum is offered globally six times a year. It was established due to the rising demand of the marker in routine diagnostics. The EQA scheme is only mandatory in Germany, if hs-CRP is part of a laboratory’s accreditation. For each survey, two blinded samples with different concentrations are sent to the participating laboratories. One sample has hs-CRP levels of around 1 mg/L (low risk) and one of around 2 mg/L (medium risk). Participants are asked to analyze the samples like normal patient samples and to report their quantitative results for hs-CRP to INSTAND’s web-based RV-Online platform (http://rv-online.instandev.de) together with information on the respective device, reagent, and method used.

As no reference measurement procedure is currently available, the consensus value of manufacturer-specific collectives, calculated using algorithm A {[International Organization for Standardization (ISO), 2023] Section C3}, serves as the target value for the evaluation of participant results and for laboratory certification. The criterion for passing the EQA was ±30% around the consensus value.

### 2.3 Data analysis and statistics

Passing rates and participant numbers were evaluated for all EQA surveys conducted between 2018 and 2023 (Supplementary Table S1). Due to the large number of EQA surveys, only the data obtained from the three annual EQAs with the largest number of participants (January, May, and October of each year) were evaluated. This resulted in 17 EQA surveys (Supplementary Table S2). The participating laboratories reported a total of 3,668 results. Results from individual participants that involved sample swaps or reporting errors were excluded from the analysis. This applied to a total of 11 datasets.

The EQA data were analyzed in a manufacturer-dependent manner. Eight manufacturer collectives (number of participants ≥8 in at least half of the surveys) were included in the analysis: Abbott (AB), Beckman Coulter (BE), Beckman Coulter-Olympus (OL), Siemens Healthineers (SI), Siemens-Dade Behring (BW), Siemens-Bayer Health (BG), Siemens-DPC Biermann (DG), and Roche Diagnostics (RO). The distributions of results are shown as box plot diagrams over time. For all boxes, the box covers the 25th percentile, the median and the 75th percentile while the whiskers stretch from the 1st quartile -1.5*(interquartile range) to the 3rd quartile +1.5*(interquartile range). The BE collective comprised two manufacturer sub-collectives (BE, OL). Therefore, they were highlighted with the same color but different filling color, since we observed multimodality in several EQA surveys. The same applies to the four manufacturer sub-collectives consolidated under SI (SI, BW, BG, and DG). Detailed information about the (sub)-collectives can be found in Supplementary Table S2. As the clinical decision limits in the literature differed greatly, the decision limits from the Clinical Laboratory Diagnostics Series by Thomas (2023), which are identical with the limits proposed by the U.S. Preventive Services Task Force (2009), were used for this evaluation. These limits are defined as low risk for CVD (below 1 mg/L), medium risk (1–3 mg/L), and high risk (3 mg/L).

The coefficients of variation (CVs) were calculated to quantify the scatter within the manufacturer collectives. Manufacturer-dependent values that scattered further than 1.5 times the inter-quartile range, the width between the 25th and 75th percentiles, were defined as outliers and excluded before the CVs were calculated. These data points are marked in orange in the raw data (Supplementary Table S2).

Harmonization of the different collectives was assessed though a longitudinal comparison of differences in median values.

Basic statistical analyses were performed using jmp 17.2.0 from SAS Institute (Cary, NC, United States). The overlay images were generated using version 2.10.8 of the Gnu image manipulation software.

## 3 Results

**FIGURE 1:**
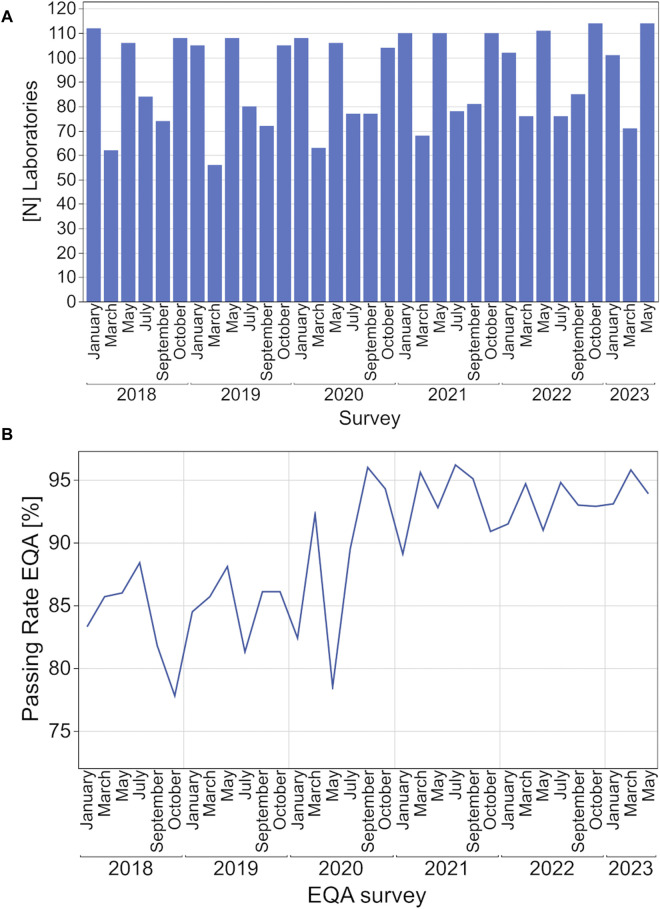
Development of participating laboratories **(A, B)** EQA passing rates for hsCRP from 2018 to 2023.

During the observed period, the number of annual participants per survey remained constant with more than 100 laboratories participating in the surveys in January, May, and October and between 56 and 81 laboratories in March, July, and September (Figure 1A). Depending on the survey, between 71% and 89% of participating laboratories were from Germany, between 4% and 23% from other EU countries and 4% to 13% from non-EU countries (Supplementary Table S1).

The passing rate for each EQA survey fluctuated between 78% and 88% from 2018 to May 2020 and rose to over 90% starting in September 2020 (Figure 1B).

**FIGURE 2:**
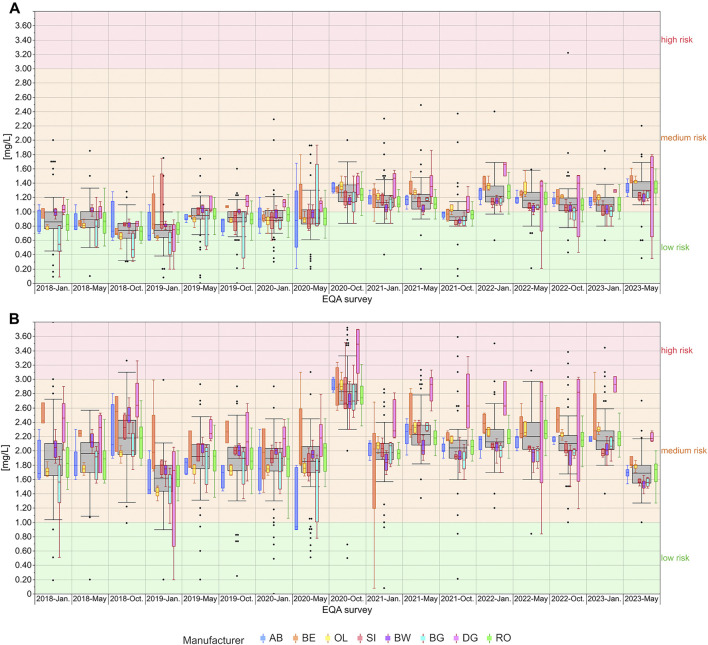
Analysis of manufacturer-dependent differences for the detection of hs-CRP in serum from 2018 to 2023 for the low concentration **(A)** and high concentration sample **(B)**. The grey boxes display all results for the respective sample, and the distributions of specific manufacturer-based collectives are illustrated as smaller, colored box plots in overlay with the total results. For all boxes, the whiskers stretch from the 1st quartile - 1.5*(interquartile range) to the 3rd quartile + 1.5*(interquartile range). OL is a sub-collective of BE, and BW, BG and DG are sub-collectives of SI, hence the the same outline but different filling. All results below 1 mg/L are considered “low risk for cardiovascular disease” (green area). Results between 1 mg/L and 3 mg/L are considered “medium risk for cardiovascular disease” (orange area) and results above 3 mg/L as “high risk for cardiovascular disease” (red area) (Thomas, 2023).

The distribution of the EQA results for hs-CRP showed manufacturer-dependent differences particularly for the DG collective, which tended to show notably higher results than the other collectives especially, but not exclusively, in the higher concentration samples (Figure 2B). In EQA samples with hs-CRP levels around the known clinical decision limits of 1 mg/L and 2 mg/L, respectively, single manufacturer collectives stayed below and/or above this decision limit. For example, for the low concentration samples used in October 2023, DG and OL mostly detected values above 1 mg/L, while SI, BW, and BG detected values clearly below 1 mg/L (Figure 2A). In other cases, the scatter of results of single collectives was large enough to span a clinical decision limit, e.g., for AB in the low concentration samples in 2018 and in January and May 2020, and for DG in the high concentration samples sent out in October 2020 as well as in May and October 2021 (Figure 2).

**FIGURE 3:**
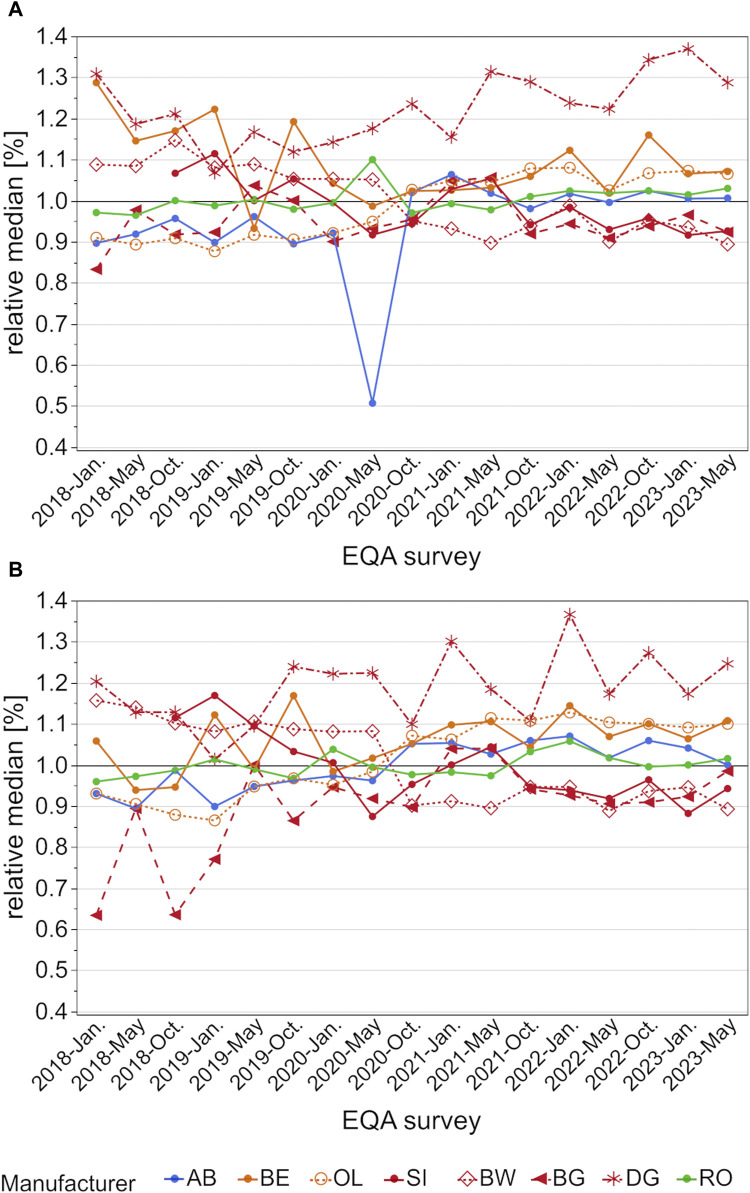
Analysis of manufacturer-dependent differences in median values for the detection of hs-CRP in serum from 2018 to 2023 for the low concentration **(A)** and high concentration sample **(B)**. All median values are normalized to the total median of the corresponding EQA scheme. OL is a sub-collective of BE, and BW, BG and DG are sub-collectives of SI, hence the same colors but different pattern.

When relative median values are compared, the DG collective had the highest median values of all observed collectives (Figure 3). Interestingly, the differences seem to increase over the observed period, especially for the higher concentration sample. Here the relative difference was over 30% in comparison to all other collectives of the SI group (Figure 3B). While BW and SI also tended to have slightly higher values, this changed in October 2020 when these groups showed lower results than the other manufacturers. At the same time, BG displayed a negative bias down to −35% for the low concentration sample, which was then reduced to the same bias as SI and BW. In general, the relative median values were up to 50% in 2018 and started to be much better aligned in October 2020, essentially only 20% apart, except for the DG collective.

**FIGURE 4:**
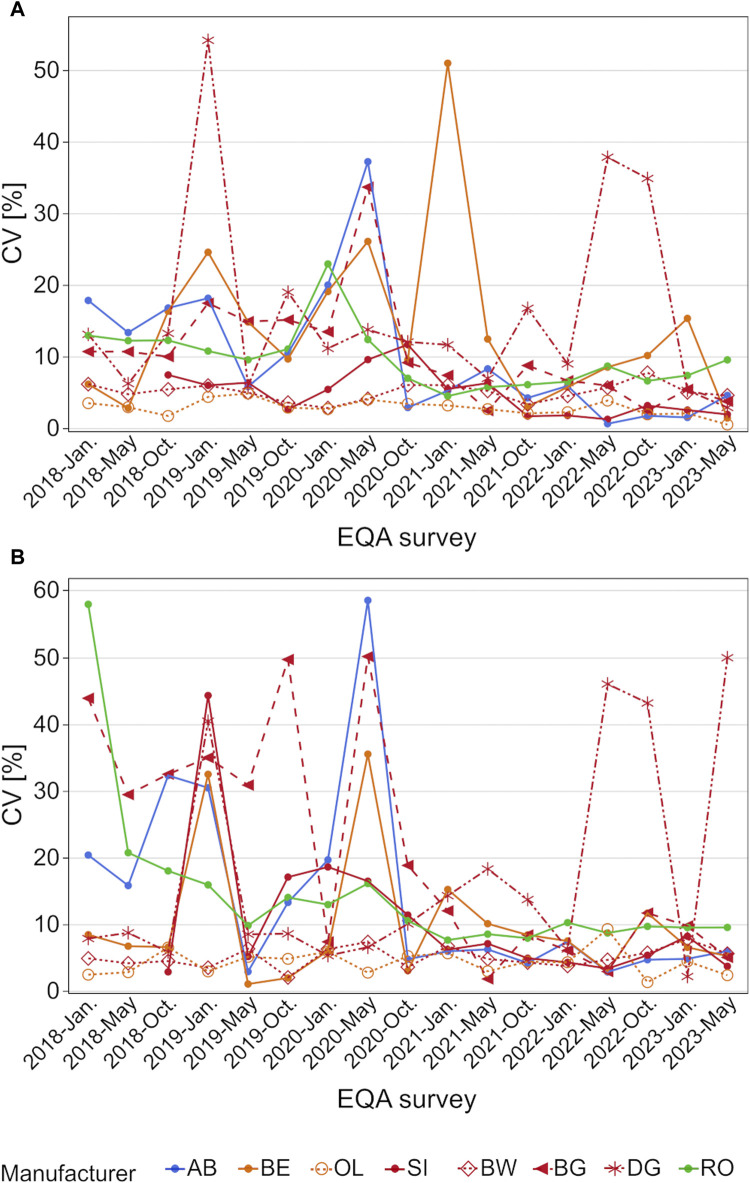
Analysis of manufacturer-dependent differences in CV for the detection of hs-CRP in serum from 2018 to 2023 for the low concentration **(A)** and high concentration sample **(B)**. OL is a sub-collective of BE, and BW, BG and DG are sub-collectives of SI, hence the same colors but different pattern.

A closer look at the scatter of results shows that many manufacturer collectives had CVs of around 50% and higher for occasional samples [e.g., BG, AB, and RO in January 2018 for the high concentration sample (Figure 4B)]. Beginning in October 2020, the CV of most collectives stayed below 10%, except for DG, which exhibited CVs of over 30% in May and October 2022 for both samples and around 50% in May 2023 for the high concentration sample (Figure 4).

## 4 Discussion

The significance of the serum-marker CRP has increased in recent decades. While concentrations >5 mg/L are a marker for tissue injury, inflammatory events and acute infection (Póvoa et al., 1998; Póvoa, 2002; Pepys and Hirschfield, 2003; Lobo, 2012), continuous moderately elevated concentrations around and above 2–3 mg/L, so called hs-CRP, have been identified as a possible risk factor for CVDs (U.S. Preventive Services Task Force, 2009; Zhang et al., 2021; Lee and Lee, 2023). This paper assessed the current quality of hs-CRP detection based on EQA data from 2018 to 2023.

Manufacturer-dependent differences as well as the scatter of results were found to decrease slightly after October 2020, when the sample material was changed from a serum pool to sera obtained from individual donors. One exception was the DG collective, whose median values increased in comparison to the other collectives, especially for the high concentration samples (Figure 3B). While the median values of the other collectives decreased from over 50% to only 20%, the DG collective exceeded the median of other manufacturer collectives by up to 35% in May 2023 (Figure 3). Promising trends were observed for the scatter of results, as the CVs of most manufacturer collectives, apart from the DG collective, stayed below 10% (Figure 4).

Our results correspond to those of several research groups that have reported similar differences between various assays. Thanabalasingham et al. (2011) observed differences between three assays from SI, labeling them methods one, two, and three. The assay from method one was used by this paper’s BG collective and the assay from method three by the BW collective. They observed higher results for the BG assay for hs-CRP concentrations >1 mg/L and higher results for the BW assay for hs-CRP concentrations below this threshold. The data from the INSTAND EQA schemes showed that in 2018 the BW collective had up to 50% higher hs-CRP median values than the BG collective, regardless of sample concentration. These differences have nearly vanished since October 2021 and now these two collectives align quite well (Figure 3), possibly due to a re-calibration of the tests.

For the collectives BE and OL, the manufacturer-dependent differences in median values observed in January 2018 decreased over time and nearly vanished from October 2020 onwards (Figure 3).

An older paper by Roberts et al. reported that the assays from AB and BE showed higher results in serum pools with more than 2 mg/L CRP than the test systems from BW (Roberts et al., 2000). In a follow-up study, the test systems from RO and OL showed comparable or slightly higher values than the system from BW (Roberts et al., 2001). Data from the EQA surveys showed that, while the difference between AB, BW, and OL had nearly vanished in the last 2 years, RO still had slightly higher median values than BW (Figure 3).

The positive trend in the harmonization between the hs-CRP assay manufacturers analyzed in this study began appearing in 2019–2020. At that time, the relative median values started to align until the observed differences were below 20% for the highest and lowest collectives, with the exception of the DG collective. Since the trend started before INSTAND changed the sample material from serum pools to single-donor samples, an influence of pooled samples on the median comparison is unlikely. Nevertheless, the change in sample material could have had an influence on the scatter results since, strikingly, the CVs stayed below 10% more often after the change in sample material (Figure 4). The occasional outliers in CV could be due to small sample sizes, e.g., the DG collective showed the highest CVs in 2022 and 2023 when fewer than ten laboratories participated in each EQA survey (Supplementary Table S2).

Another reason for the good harmonization observed in this paper is the presence of a certified reference material for hs-CRP: ERM DA474/IFCC. The CRP value for this standard was assigned using ERM-DA470 as a calibrant, which is traceable to the WHO International Standard 85/506 (Hanisch et al., 2011). The follow-up standard for ERM-DA470, ERM-DA470k/IFCC, was unsuitable for the certification of CRP due to a roughly 20% loss of CRP in the lyophilized standard preparation when compared to frozen material, as measured by routine immunoassays (Zegers et al., 2010).

Interestingly, SI reassigned calibrator lots for their Advia (BG collective) and Atellica (SI collective) hs-CRP assays from ERM-DA470 to ERM DA474/IFCC as they observed a positive bias of approximately 15% for patient samples and quality control material when compared to ERM DA474/IFCC (Siemens Healthineers, 2020a; Siemens Healthineers, 2020b). In the meantime, several RO hs-CRP assays still state their traceability to the ERM-DA470 standard preparation (Roche Diagnostics GmbH, 2023a; Roche Diagnostics GmbH, 2023b).

These differences in the traceability of calibrators could be one factor in why the BW and the SI collectives showed such a clear drop in relative median results around May and October 2020. While the SI collective rose once again for a short time, both collectives showed almost identical results beginning in October 2021. The RO collective exhibited relative median values that were around 10% higher than those of BW and SI starting in October 2021, but they aligned well in May 2023 for the higher concentration sample (see Figure 3).

Systems from BW [e.g., (de Lemos et al., 2017; Tunstall-Pedoe et al., 2017; Khera et al., 2018; Lee et al., 2018; Zhang et al., 2021)] and RO [e.g., (Petersson et al., 2009; Eugen-Olsen et al., 2010)] were most frequently deployed in several meta-analyses. The observed differences in the INSTAND EQA surveys clearly show that the observed assay variability could have a significant impact on the “real-life” CVD risk classification for patients, despite the relatively good harmonization (Figure 2). For example, for the high concentration samples analyzed in May and October 2022 (Figure 2B), 75% of laboratories using BW reported results of <2 mg/L, while over 75% of RO laboratories reported results of >2 mg/L for the same patient. Therefore, meta-analyses that compile data from different clinical studies should not only be stratified by study population and research question, but also by hs-CRP assay to ensure valid data aggregation and interpretation.

One limitation of this study is that it is not possible to assess whether the changes in median results, especially in the sub-collectives of SI, are due to the recalibration of their calibrators or due to the change in sample material from serum pools to single-donor samples. But since a positive trend for harmonization was observed before the change in sample material, it is unlikely that the effect is solely based on that switch. Furthermore, some of the collectives were quite small, which could bias the CV calculation.

The results from this analysis clearly show the high importance of a well-tailored diagnosis and treatment policy in CVD patients. However, while huge efforts have been made to raise the level of assay harmonization for this marker to the current level, new complications appear on the horizon. At a recent JCTLM workshop, data were presented on newly developed primary pure candidate substances and secondary certified reference materials (CRMs). Furthermore, new reference measurement procedures indicate that clinical samples measured with procedures that are calibrated with the CRMs mentioned might clearly differ from results measured by the immunoassays currently calibrated to the existing standard materials. However, the possible influence of such new CRMs or RMPs of higher order on the measurement of CRP and hs-CRP still requires further assessment (Miller et al., 2023).

## 5 Conclusion

While the harmonization of hs-CRP assays is quite good, the observed bias in the EQA surveys could still lead to a clinical misclassification in the case of risk stratification for CVDs under real life conditions. Although our data do not provide any insight on the dimension of this risk, it is clear that hs-CRP should not be used as a single marker for risk stratification and longitudinal measurements of the same patient and should always be done in the same device. For a better future harmonization, new developments in reference materials and reference measurement procedures for CRP and hs-CRP need to be carefully observed. Especially without a proper reference measurement procedure it is currently impossible to give any recommendations for or against an assay for the detection of hs-CRP as well as its use in a CVD risk score. But meta-analysis of different risk-score publications should stratify their analysis by assay to account for the observed manufacturer-specific differences observed in this paper.

## Data Availability

The original contributions presented in the study are included in the article/Supplementary Material, further inquiries can be directed to the corresponding author.

## References

[B1] Ahmad A. Lim L. L. Morieri M. L. Tam C. H. Cheng F. Chikowore T. (2024). Precision prognostics for cardiovascular disease in Type 2 diabetes: a systematic review and meta-analysis. Commun. Med. (Lond) 4 (1), 11. 10.1038/s43856-023-00429-z 38253823 PMC10803333

[B2] Australian Chronic Disease Prevention Alliance (2023). Australian Guideline for assessing and managing cardiovascular disease risk. 10.18773/austprescr.2024.014 PMC11081742 38737366

[B3] CDC (2021). Health topics – heart disease and heart attack. Available at: https://www.cdc.gov/policy/polaris/healthtopics/heartdisease/index.html.

[B4] Charoud-Got J. Zegers I. Rzychon M. Centre, Joint Research, Materials, Institute for Reference and Measurements (2009). Certification of C-reactive protein in reference material ERM-DA472/IFCC certified reference materials ERM-DA472/IFCC. Publications Office.

[B5] de Lemos J. A. Ayers C. R. Levine B. D. deFilippi C. R. Wang T. J. Hundley W. G. (2017). Multimodality strategy for cardiovascular risk assessment: performance in 2 population-based cohorts. Circulation 135 (22), 2119–2132. 10.1161/CIRCULATIONAHA.117.027272 28360032 PMC5486874

[B6] Deutsche Gesellschaft für Allgemeinmedizin und Familienmedizin e.V (2017). Hausärztliche Risikoberatung zur kardiovaskulären Prävention. S3-Leitlinie, AWMF.

[B7] Eugen-Olsen J. Andersen O. Linneberg A. Ladelund S. Hansen T. W. Langkilde A. (2010). Circulating soluble urokinase plasminogen activator receptor predicts cancer, cardiovascular disease, diabetes and mortality in the general population. J. Intern Med. 268 (3), 296–308. 10.1111/j.1365-2796.2010.02252.x 20561148

[B8] European Society of Cardiology (2023). Price tag on cardiovascular disease in Europe higher than entire EU budget.

[B9] Hanisch K. Zegers I. Schimmel H. Boulo S. Emons H. Trapmann S. (2011). The certification of the mass concentration of C-reactive protein in human serum – certified reference material ERM®-DA474/IFCC. Publications Office.

[B10] Heidemann C. Scheidt-Nave C. Beyera A.-K. Baumert J. Thamm R. Maier B. (2021). Gesundheitliche Lage von Erwachsenen in Deutschland-Ergebnisse zu ausgewählten Indikatoren der Studie GEDA 2019/2020-EHIS. J. Health Monit. 6 (3), 3–27. 10.25646/8459 PMC8734117 35146314

[B11] International Organization for Standardization [ISO] (2023). Konformitätsbewertung–Allgemeine Anforderungen an die Kompetenz von Anbietern von Eignungsprüfungen (ISO/IEC 17043:2023); Deutsche und Englische Fassung EN ISO/IEC 17043:2023. DDIfNe V.

[B12] Khera A. Budoff M. J. O’Donnell C. J. Ayers C. A. Locke J. de Lemos J. A. (2018). Astronaut cardiovascular Health and risk modification (Astro-CHARM) coronary calcium atherosclerotic cardiovascular disease risk calculator. Circulation 138 (17), 1819–1827. 10.1161/CIRCULATIONAHA.118.033505 30354651

[B13] Lee D. Y. Rhee E. J. Chang Y. Sohn C. I. Shin H. C. Ryu S. (2018). Impact of systemic inflammation on the relationship between insulin resistance and all-cause and cancer-related mortality. Metabolism 81, 52–62. 10.1016/j.metabol.2017.11.014 29191456

[B14] Lee H. S. Lee J. H. (2023). Early elevation of high-sensitivity C-reactive protein as a predictor for cardiovascular disease incidence and all-cause mortality: a landmark analysis. Sci. Rep. 13 (1), 14118. 10.1038/s41598-023-41081-w 37644061 PMC10465521

[B15] Li Y. Zhong X. Cheng G. Zhao C. Zhang L. Hong Y. (2017). Hs-CRP and all-cause, cardiovascular, and cancer mortality risk: a meta-analysis. Atherosclerosis 259, 75–82. 10.1016/j.atherosclerosis.2017.02.003 28327451

[B16] Libby P. Ridker P. M. Maseri A. (2002). Inflammation and atherosclerosis. Circulation 105 (9), 1135–1143. 10.1161/hc0902.104353 11877368

[B17] Lin J. S. Evans C. V. Johnson E. Redmond N. Coppola E. L. Smith N. (2018). Nontraditional risk factors in cardiovascular disease risk assessment: updated evidence report and systematic review for the US preventive Services Task Force. Jama 320 (3), 281–297. 10.1001/jama.2018.4242 29998301 PMC13564044

[B18] Lobo S. M. (2012). Sequential C-reactive protein measurements in patients with serious infections: does it help? Crit. Care 16 (3), 130. 10.1186/CC11347 22731851 PMC3580631

[B19] Miller W. G. Panteghini M. Wielgosz R. (2023). Implementing metrological traceability of C-reactive protein measurements: consensus summary from the joint committee for traceability in laboratory medicine workshop. Clin. Chem. Lab. Med. 61 (9), 1558–1560. 10.1515/cclm-2023-0498 37253275

[B47] Müller I. Besier S. Hintereder G. Brade V. Hunfeld K. (2009). Zur Qualität der bakteriologischen Infektionsserologie in Deutschland: eine Metaanalyse der infektionsserologischen Ringversuche des Jahres 2006—Beitrag der Qualitätssicherungskommission der DGHM. GMS Z Forder Qualitatssich Med. Lab. 1, 1–21.

[B20] Musunuru K. Kral B. G. Blumenthal R. S. Fuster V. Campbell C. Y. Gluckman T. J. (2008). The use of high-sensitivity assays for C-reactive protein in clinical practice. Nat. Clin. Pract. Cardiovasc Med. 5 (10), 621–635. 10.1038/ncpcardio1322 18711404 PMC2639398

[B21] Oude Wolcherink M. J. Behr C. M. Pouwels X. Doggen C. J. M. Koffijberg H. (2023). Health economic research assessing the value of early detection of cardiovascular disease: a systematic review. Pharmacoeconomics 41 (10), 1183–1203. 10.1007/s40273-023-01287-2 37328633 PMC10492754

[B22] Pepys M. B. Hirschfield G. M. (2003). C-reactive protein: a critical update. J. Clin. investigation 111 (12), 1805–1812. 10.1172/JCI18921 PMC161431 12813013

[B23] Petersson U. Ostgren C. J. Brudin L. Nilsson P. M. (2009). A consultation-based method is equal to SCORE and an extensive laboratory-based method in predicting risk of future cardiovascular disease. Eur. J. Cardiovasc Prev. Rehabil. 16 (5), 536–540. 10.1097/HJR.0b013e32832b1833 19357517

[B24] Piepoli M. F. Hoes A. W. Agewall S. Albus C. Brotons C. Catapano A. L. (2016). 2016 European guidelines on cardiovascular disease prevention in clinical practice: the sixth joint Task Force of the European society of Cardiology and other societies on cardiovascular disease prevention in clinical practice (constituted by representatives of 10 societies and by invited experts)Developed with the special contribution of the European association for cardiovascular prevention and rehabilitation (EACPR). Eur. Heart J. 37 (29), 2315–2381. 10.1093/eurheartj/ehw106 27222591 PMC4986030

[B25] Póvoa P. (2002). C-reactive protein: a valuable marker of sepsis. Intensive Care Med. 28 (3), 235–243. 10.1007/s00134-002-1209-6 11904651

[B26] Póvoa P. Almeida E. Moreira P. Fernandes A. Mealha R. Aragão A. (1998). C-reactive protein as an indicator of sepsis. Intensive Care Med. 24 (10), 1052–1056. 10.1007/s001340050715 9840239

[B27] Ridker P. M. Danielson E. Fonseca F. A. Genest J. Gotto A. M. Jr. Kastelein J. J. (2008). Rosuvastatin to prevent vascular events in men and women with elevated C-reactive protein. N. Engl. J. Med. 359 (21), 2195–2207. 10.1056/NEJMoa0807646 18997196

[B28] Ridker P. M. Everett B. M. Thuren T. MacFadyen J. G. Chang W. H. Ballantyne C. (2017). Antiinflammatory therapy with canakinumab for atherosclerotic disease. N. Engl. J. Med. 377 (12), 1119–1131. 10.1056/nejmoa1707914 28845751

[B29] Roberts W. L. Moulton L. Law T. C. Farrow G. Cooper-Anderson M. Savory J. (2001). Evaluation of nine automated high-sensitivity C-reactive protein methods: implications for clinical and epidemiological applications. Part 2. Clin. Chem. 47 (3), 418–425. 10.1093/clinchem/47.3.418 11238291

[B30] Roberts W. L. Sedrick R. Moulton L. Spencer A. Rifai N. (2000). Evaluation of four automated high-sensitivity C-reactive protein methods: implications for clinical and epidemiological applications. Clin. Chem. 46 (4), 461–468. 10.1093/clinchem/46.4.461 10759469

[B31] Roche Diagnostics GmbH (2023a). “Cardiac C-reactive protein (latex) high sensitive (cobas c 111).” V 9.0 English. Available at: https://elabdoc-prod.roche.com/eLD/api/downloads/fbc6a02f-8a83-ee11-2291-005056a71a5d?countryIsoCode=pi.

[B32] Roche Diagnostics GmbH (2023b). “Cardiac C-reactive protein (latex) high sensitive (cobas c 303, cobas c 503).” V 9.0 English. Available at: https://elabdoc-prod.roche.com/eLD/api/downloads/fbc6a02f-8a83-ee11-2291-005056a71a5d?countryIsoCode=pi.

[B33] Romero-Cabrera J. L. Ankeny J. Fernández-Montero A. Kales S. N. Smith D. L. (2022). A systematic review and meta-analysis of advanced biomarkers for predicting incident cardiovascular disease among asymptomatic middle-aged adults. Int. J. Mol. Sci. 23 (21), 13540. 10.3390/ijms232113540 36362325 PMC9656299

[B34] Shah T. Casas J. P. Cooper J. A. Tzoulaki I. Sofat R. McCormack V. (2009). Critical appraisal of CRP measurement for the prediction of coronary heart disease events: new data and systematic review of 31 prospective cohorts. Int. J. Epidemiol. 38 (1), 217–231. 10.1093/ije/dyn217 18930961 PMC2639366

[B35] Siemens Healthineers (2020a). Reassignment of the ADVIA® chemistry CardioPhase high sensitivity C-reactive protein (hsCRP) calibrator lots 484707 and 516407. Tarrytown, NY 10591.

[B36] Siemens Healthineers (2020b). Reassignment of the Atellica CH high sensitivity C-reactive protein (hsCRP) calibrator lots 484721 and 516427. Tarrytown, NY 10591.

[B37] Statistisches Bundesamt (2022). Krankheitskosten pro Kopf gleichen sich zwischen Männern und Frauen weiter an.

[B38] Stevenson E. Walsh C. Thomas S. (2023). EQA: there’s not a glitch in the matrix. Investigation of CRP bias on the Roche Cobas c701. Ann. Clin. Biochem. 60 (5), 349–352. 10.1177/00045632231169151 37015888

[B39] Thanabalasingham G. Shah N. Vaxillaire M. Hansen T. Tuomi T. Gašperíková D. (2011). A large multi-centre European study validates high-sensitivity C-reactive protein (hsCRP) as a clinical biomarker for the diagnosis of diabetes subtypes. Diabetologia 54 (11), 2801–2810. 10.1007/s00125-011-2261-y 21814873

[B40] Thomas L. (2023). Atherosclerosis - development of atherosclerosis. Clinical laboratory diagnostics.

[B41] Tunstall-Pedoe H. Peters S. A. E. Woodward M. Struthers A. D. Belch J. J. F. (2017). Twenty-year predictors of peripheral arterial disease compared with coronary heart disease in the scottish heart Health extended cohort (SHHEC). J. Am. Heart Assoc. 6 (9), e005967. 10.1161/JAHA.117.005967 28923990 PMC5634266

[B42] U.S. Preventive Services Task Force (2009). Using nontraditional risk factors in coronary heart disease risk assessment: U.S. Preventive Services Task Force recommendation statement. Ann. Intern Med. 151 (7), 474–482. 10.7326/0003-4819-151-7-200910060-00008 19805770

[B43] Visseren F. L. J. Mach F. Smulders Y. M. Carballo D. Koskinas K. C. Bäck M. (2022). 2021 ESC Guidelines on cardiovascular disease prevention in clinical practice: developed by the Task Force for cardiovascular disease prevention in clinical practice with representatives of the European Society of Cardiology and 12 medical societies with the special contribution of the European Association of Preventive Cardiology (EAPC). Rev. Esp. Cardiol. Engl. Ed. 75 (5), 429. 10.1016/j.rec.2022.04.003 35525570

[B44] WHO (2023). Noncommunicable diseases. Available at: https://www.who.int/news-room/fact-sheets/detail/noncommunicable-diseases.

[B48] Wojtalewicz N. Schellenberg I. Hunfeld K. P. (2019). Evaluation of INSTAND e.V.’s external quality assessment for C-reactive protein and procalcitonin. PLoS One 14 (8), e0221426. 31419260 10.1371/journal.pone.0221426 PMC6697325

[B45] Zegers I. Schreiber W. Linstead S. Lammers M. McCusker M. Muñoz A. (2010). Development and preparation of a new serum protein reference material: feasibility studies and processing. Clin. Chem. Lab. Med. 48 (6), 805–813. 10.1515/CCLM.2010.166 20374041

[B46] Zhang W. Speiser J. L. Ye F. Tsai M. Y. Cainzos-Achirica M. Nasir K. (2021). High-Sensitivity C-reactive protein modifies the cardiovascular risk of lipoprotein(a): multi-ethnic study of atherosclerosis. J. Am. Coll. Cardiol. 78 (11), 1083–1094. 10.1016/j.jacc.2021.07.016 34503676 PMC8444216

